# Peer Review of “COVID-19 National Football League (NFL) Injury Analysis: Follow-Up Study”

**DOI:** 10.2196/55900

**Published:** 2024-02-13

**Authors:** ­ Anonymous

**Keywords:** COVID-19, injury, prevalence, adaptation, sports medicine, follow-up, training, football, epidemiology, sport, athlete, athletic, injuries

*This is the peer-review report for “COVID-19 National Football League (NFL) Injury Analysis: Follow-Up Study.”*

## Round 1 Review

This paper, “COVID-19 NFL Injury Prevalence Analysis, A Follow-Up Study” [1], is an interesting read. The conclusions drawn in the paper are supported by the data. All the related works are cited appropriately. The limitations of the presented study are discussed appropriately.

My only concern is about the 2 histograms. The authors should make these histograms more clear, readable, and engaging. Use standard deviation or error bars wherever applicable.

## References

[1] Puga TB, Schafer J, Thiel G (2024). COVID-19 National Football League (NFL) injury analysis: follow-up study. JMIRx Med.

